# Metabolic distress in lipid & one carbon metabolic pathway through low vitamin B-12: a population based study from North India

**DOI:** 10.1186/s12944-018-0748-y

**Published:** 2018-04-25

**Authors:** Kallur Nava Saraswathy, Shipra Joshi, Suniti Yadav, Priyanka Rani Garg

**Affiliations:** 1 Department of Anthropology, University of Delhi, 110007, Delhi, India; 2 Public health foundation of India, plot no. 47, sector 44, Delhi, NCR 122002 India

**Keywords:** Lipid, Homocysteine, Vitamin B-12, Metabolic pathway, Under-nutrition, Population

## Abstract

**Background:**

Dyslipidemia and hyper-homocysteinemia are the major independent risk factors of cardio vascular disease. Deficiency of folate and vitamin B-12 are associated with both hyper-homocysteinemia and dyslipidemia. The aim of the study is to evaluate the relationship of homocysteine and its associated dietary determinant levels (Folate and Vitamin B-12) with lipids and obesity parameters (WC, BMI, WHR) in North Indian population.

**Methods:**

The participants were recruited under a major government funded project through household survey covering 15 villages of Haryana, India. Participants were both males and females, between age group 30–65 years, from a north Indian community. Initially 1634 individuals were recruited, of which 1374 were considered for analysis as they were not found to be on any kind of medication for high blood pressure, CAD, diabetes or any other disorder, and had no missing data. 5 mL of intravenous blood sample was collected after obtaining written informed consent from the participants. Homocysteine, folate and vitamin B12 levels were estimated through Immulite 1000 by chemi-luminescence technique. Triglyceride, total cholesterol and HDL-C were estimated by spectrophotometry technique using commercial kits. The values for LDL and VLDL were calculated using Friedwald’s equation. Height, weight, waist circumference (WC), hip circumference (HC) was measured over light clothing. Statistical analysis for data was performed using SPSS 16.0 version.

**Results:**

All the lipid indices, except HDL, showed a trend of negative correlation with homocysteine after controlling for confounders, though not significant. No association was found between obesity (WC, BMI, WHR) and homocysteine in the present study. Vitamin B-12 deficiency was significantly associated with both hyper-homocysteinemia and low HDL**.** Folate was found to have significantly reduced risk for high TC & LDL.

**Conclusions:**

The “hcy-lipid” hypothesis does not seem to be complementing in the present studied population. The population is vulnerable to severe under-nutrition due to the association of vitamin B-12 with HDL, leading to metabolic disturbance in both the pathways; lipid and one carbon metabolic pathway. Co-factors such as ethnicity, cultural practices, and lifestyle & dietary habits must be considered while making public health policies to control diseases.

**Electronic supplementary material:**

The online version of this article (10.1186/s12944-018-0748-y) contains supplementary material, which is available to authorized users.

## Background

The most common and highest population attributable traditional risk for CVDs is dyslipidemia [1], its prevalence ranges from 10 to 73% among Asian Indians [2]. Abnormality in any one of the lipid indices; TC, TG, HDL, LDL, VLDL is considered as dyslipidemic. Dyslipidemia is a phenomenon associated with both genetic or environmental factors such as eating habits coupled with less physical activity [3–5], tobacco and alcohol consumption.

On the other hand, Hcy is a metabolite in one carbon metabolic pathway, and higher level of Hcy (≥15 μmol/L) was found to be contributing to the development and progression of CVD’s and various other complex disorders [6–8]. Its prevalence is in the range of 52–84% in different population groups in India [9–11]. HHcy disturbs the phospholipid metabolism by affecting the assembly or secretion of VLDL, leading to dyslipidemia [12]. Hcy mediated enhanced lipid peroxidation and generation of free radicals results in inflammation and acute endothelial dysfunction which accelerates atherosclerotic process predisposing to cardiovascular disease [13].The lipid and one carbon metabolic pathway are connected via an intermediate methyl donor, S-Adenosyl methionine (SAM). SAM has dual role, first it donates methyl groups to S-adenosylhomocysteine (SAH) for proper functioning of one carbon metabolic pathway and second is to donate 3 methyl groups to phosphatidylethanolamine (PE) for the production of 30% of phosphatidylcholine (PC) via PEMT pathway, which is responsible for 50% of plasma hcy levels. Synthesis of PC is important for phospholipid metabolism as PC is a component of VLDL particle and inadequate PC can cause accumulation of fat and cholesterol. PC can also be synthesized upto 70% by a second pathway which is CDP-Choline or Kennedy pathway, and this biological reaction requires either choline in dietary form or can be derived from catabolism of PC. Kennedy pathway and homocysteine metabolism are linked via choline [12]. HHcy and dyslipidemia, both are the independent risk factors of CVDs but the co-existence of both HHcy & dyslipidemia is likely to multiply the burden of CVDs. Folate and vitamin B-12 (methyl donors) are the essential components of Hcy metabolic pathway and deficiency of these methyl donors are not only associated with HHcy but also dyslipidemia as reported in studies [10].

Multiple researchers have examined the relationship between HHcy and dyslipidemia; however the results are inconsistent [14] in case-control studies as well as in population studies. Hence, it is important to explore the “hcy-lipid” relationship in Indian population to help control the risks associated with the co-existence of HHcy & dyslipidemia, which is a leading cause of CVDs.

A preliminary work done on the prevalence rates of CVD risk factors in the studied population group was already reported in a previous manuscript (communicated). The presently studied population from North Indian followed a lacto vegetarian diet, which is cardio-protective, in spite of that the prevalence rate of HHcy and dyslipidemia in the population was found to be reasonably high. More than 2/3rd of the present studied population had high Hcy levels (70%). The most prevalent lipid abnormality in the studied population was found to be low HDL-C (43%), while other dyslipidemic indices ranged from 22% (abnormal LDL-C) to 28% (Total Cholesterol). On the other hand prevalence of under-nutrition in terms of low dietary intake of vitamin B-12 and folate were found to be 57% and 32%, respectively in the studied population.

In view of the high prevalence rates of the risk factors, it is expected that under-nutrition in terms of low vitamin B-12, folate and HDL might be the cause of high Hcy levels which may further increase the levels of bad lipids (TC,TG, LDL) in this population, affecting two major metabolic pathways (Hcy and lipid pathway). Thus the present study was conducted with the aim to evaluate the relationship of Hcy and its associated dietary determinants (Folate and Vitamin B-12) with lipids and obesity parameters (WC, BMI, WHR), in a North Indian population.

## Methods

### Study design and area

The present study was a cross-sectional study, conducted in the Palwal district of Haryana (North India). Palwal is located near developed cities of India i.e., Delhi and Gurgaon. Palwal district has both agricultural and commercial areas. Delhi-Mathura highway (NH2) crosses the Palwal district of Haryana making it more commercial or industrialized area.

### Data collection

Pretested and modified interview schedule was administered on recruited individuals. Data on age, gender, education status, occupation, lifestyle (smoking) and physical activity were collected from all the participants. Smokers were those individuals presently smoking beedi/cigarette and had at least 15 years of smoking history. Physical activity was self reported as active and sedentary. Those who followed routine walking, standing, physically strenuous work were considered as active participants and those with predominantly sedentary routine were considered as sedentary participants. Occupation was majorly categorized as agricultural workers, government employees, business persons, and others (students, retired persons, dependents). All these factors were included in the study to check for confounders. Anthropometric measurements such as height, weight, waist circumference (WC), hip circumference (HC) were measured over light clothing [15].

### Cut-offs used for obesity parameters

Abdominal obesity was defined as WC ≥90 cm for men and ≥ 80 cm for women [16]. High WHR was defined as > 0.90 cm for men and > 0.80 cm for women [17]. High BMI (kg/m^2^), i.e., overweight and obese were defined as BMI 23–24.9 kg/m^2^ and BMI ≥ 25 kg/m^2.^ respectively whereas normal BMI was defined as BMI 18–22.9 kg/m^2.^as per Asian guidelines.

### Blood sample collection

Intravenous blood sample (5 mL), after overnight fasting for at-least 12 h, was collected by trained personnel. Serum and plasma separation was done within 3 h of collection and stored at − 80 °C.

Lipid parameters i.e. triglyceride (TG), total cholesterol (TC) and HDL-C were estimated by spectrophotometry technique (Dialab instrument) using commercial kits (Randox Laboratories Ltd.). The value for LDL was calculated using Friedwald’s equation and VLDL was calculated as one-fifth of TG level. Homocysteine, folate and vitamin B-12 levels were estimated through Immulite 1000 Analyser (Siemens Diagnostic products, USA) by chemi-luminescence technique as per the manufacturer’s instruction. As per the specifications of the manufacturer, the calibrator range for Hcy was 2–50 μmol/L. For every batch of samples, one sample was reanalyzed for quality check for all biochemical tests, and samples with co-efficient of variation less than 20% was used in the analysis. All biochemical estimations were performed at All India Institute of Medical Sciences (AIIMS), New Delhi.

### Cut-offs used for biochemical parameters

NCEP guidelines were used to estimate dyslipidemia. High TC was defined as > 200 mg/dL, high TG as ≥150 mg/dL, low HDL as < 40 for men and < 50 for women (mg/dL), high LDL as > 130 mg/dL, high VLDL as > 30 mg/dL. High levels of Hcy was defined as ≥15 μmol/L and vitamin B-12 deficiency as ≤220 pg/ml and folate deficiency as < 3 ng/ml [18].

### Recruitment of subjects

The participants were recruited, under a major government funded project, which also included genetic analysis. It is because of this that the major criterion for recruitment was that the individuals will be unrelated upto first cousin. Recruitment of participants was done through household survey of 15 villages of Haryana. The population was considered to be from north Indian gene pool and speaks a language which is categorized as Indo-European linguistic group. They follow caste endogamy, village and surname exogamy, as per the data captured using pretested and modified interview schedules.

Total 1634 individuals were recruited initially, but 260 participants were excluded as they had missing data and/or they were found to be on medication for high blood pressure, CAD, diabetes, or any other disorders. Finally, 1374 were found to be meeting the criteria that none were on any kind of medication/supplementation, and were unrelated samples within age group 30-65 years with no missing data. Of 1374 subjects included in the present study, 975 subjects with high Hcy were considered as cases and 399 subjects with normal Hcy were considered as controls.

### Statistical analysis

Statistical analysis for data was performed using SPSS 16.0 version. Normality test was performed for all the variables. For skewed variables, median levels were considered and Mann-Whitney U-test of significance was performed. Outliers were checked for the variables through descriptive statistics-explore and were removed. Chi square test was used for frequency distribution for categorical variables. Spearman correlation and partial correlation after adjustment for confounders was performed to determine the relationship between the studied variables. Multivariate logistic regression analysis was used to determine risk after adjustment with confounders. A significance level of 5% was used for all the statistical tests.

### Ethical considerations

The study was approved by the Ethical Committee of Department of Anthropology, University of Delhi. The objectives were clearly explained to the participants of all the households and local authorities. Informed written consent of all the participants was taken prior to recruitment. Participants were informed about the data and blood sample collection in the consent form.

## Results

The comparison of general characteristics between cases (hyper-homocysteinemia) and controls (normal homocysteine) showed that the median age was significantly higher (48.00 years) among cases as compared to controls (45.00 years) (Table 1). Male sex and smokers were found to be significantly higher in cases as compared to that of controls.

**Table 1:** General characteristics of cases (hyper-homocysteinemia) and controls (normal homocysteine)

General characteristics	High Hcy	Normal Hcy	Chi-Square, *p*-value
Age (Median, in years)	48.00 (40.00–55.00)	45.00 (38.00–53.00)	*0.000* (Mann whitney test)
Smokers (%)	56.3	45.3	*0.000*
Non smokers (%)	43.7	54.7	
Gender			*0.000*
Males (%)	37.6	18	
Females (%)	62.4	82	
Education			*0.001*
Illiterate (%)	53.8	64.5	
Literate (%)	46.2	35.5	
Physical Activity			0.685
Sedentary (%)	15.9	17.0	
Active (%)	84.1	83.0	
Occupation			0.373
Government employees + Business persons + others (%)	11.9	10.1	
Agricultural worker (%)	88.1	89.9	

*P*-value at ≤0.05 level are italicized

Education was found to be significantly different among the two groups (Table 1). However, with respect to physical activity and occupation, the two groups were found to be similar. Hence, the major confounders for the present study were age, gender, education and smoking.

All the selected biochemical and anthropometric variables, i.e.; homocysteine, lipids, vitamin B-12, folate, WC, BMI, WHR were not normally distributed, so median values were considered for the present study. The median levels of all the selected lipid indices and anthropometric variables (WC, & BMI) were found to be similarly distributed among cases and controls, except for WHR. Hyper-homocysteinemic females were found to have significantly high WHR as compared to controls (Table 2).

**Table 2:** Median (IQR) levels of lipid and obesity indices among cases and controls

CVD risk factors	High Hcy (Median, IQR)	Normal Hcy (Median, IQR)	Mann-Whitney test (*P*-value)
TC	172.42 (146.31–203.90)	174.06 (147.94–202.00)	0.997
TG	99.45 (70.21–139.33)	101.32 (72.47–144.80)	0.318
HDL (Males)	46.78 (37.44–56.57)	43.36 (35.68–52.39)	0.109
HDL (Females)	50.56 (42.24–58.56)	50.24 (41.60–58.94)	0.786
LDL	97.25 (74.62–124.58)	99.18 (78.79–120.38)	0.951
VLDL	19.86 (14.03–27.51)	20.23 (14.47–28.92)	0.290
WC (Males)	84.30 (77.00–94.00)	85.25 (76.30–91.55)	0.816
WC(Females)	80.50 (72.00–89.00)	79.50 (71.35–88.35)	0.223
BMI	21.09 (18.71–24.13)	21.37 (19.02–24.21)	0.226
WHR (Males)	0.94 (0.88–0.99)	0.92 (0.87–0.97)	0.137
WHR (Females)	0.88 (0.82–0.93)	0.86 (0.81–0.92)	*0.016*
FOL	3.42 (2.38–4.53)	3.70 (2.40–5.14)	*0.008*
VIT B-12	214.00 (181.00–272.50)	239.50 (191.00–324.75)	*0.000*

*P*-value at ≤0.05 level are italicized

Considering nutritional parameters, the median levels of both folate and vitamin B-12 were significantly lower in cases as compared to controls (Table 2).

Spearman correlation and partial correlation (adjusted for age, gender, education, smoking) were performed to determine the relation of Hcy with lipids, and anthropometric variables. A weak positive correlation was found between Hcy and WC and also with WHR (Additional file 1) in spearman correlation, however, it was not persistent in partial correlation analysis after adjusted with confounders. Hcy was found to be negatively correlated to both folate and vitamin B-12 (*p* value ≤0.05) in spearman correlation as well as in partial correlation analysis (Additional file 1).

**Fig. 1:**
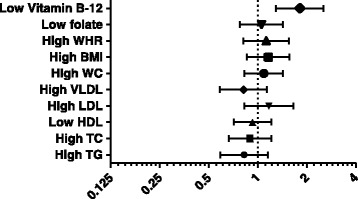
Multivariate logistic regression analysis of hcy with lipids, obesity indices and vitamins. Adjusted for confounders such as age, gender, smoking, education

Multivariate logistic regression analysis was performed for the association of Hcy with lipids, obesity indices and vitamins. Among all variables, only vitamin B12 deficiency showed more than 1 fold significant increased risk for HHcy (Fig. 1).

Distribution of general characteristics between vitamin B12 normal & deficient groups and folate normal & deficient groups revealed that males were found to be more deficient among individuals with low vitamin B-12 and folate. Gender was the major confounder. All the other demographic characteristics such as age, education, smoking, physical activity and occupation were similar in normal vitamin B-12 and low vit B-12 individuals and/or in normal folate and low folate individuals (Additional file 2: Table S1). To understand if micro-nutrient deficiencies (vitamin B-12 and folate) were the reason for dyslipidemia and anthropometric obesity, Mann-Whitney test was performed (Table 3). Vitamin B-12 median levels were found to be significantly low in subjects with low HDL (*p* ≤ 0.05). Moreover, a different trend was observed w.r.t folate; where median levels of folate were found to be significantly increased among individuals with high TC and LDL (Table 3).

**Table 3:** Median (IQR) levels of B-vitamins among individuals with normal & abnormal lipids and anthropometric variables

	Vitamin B-12 (pg/mL)	Mann-whitney test *p*-value	Folate (ng/mL)	Mann-whitney test *p*-value
Normal BMI	220.00 (185.00–291.00)	0.714	3.48 (2.32–4.62)	0.651
High BMI	217.00 (180.00–292.00)		3.45 (2.44–4.67)	
Normal WC	220.00 (185.50–280.50)	0.919	3.46 (2.36–4.76)	0.925
High WC	220.00 (183.00–293.75)		3.45 (2.40–4.65)	
Normal WHR	218.00 (186.00–273.25)	0.846	3.40 (2.39–4.58)	0.518
High WHR	220.50 (184.00–294.00)		3.47 (2.39–4.74)	
Normal TC	220.00 (185.00–278.00)	0.288	3.41 (2.24–4.62)	*0.014*
High TC	222.00 (181.00–308.00)		3.60 (2.74–4.91)	
Normal TG	221.00 (186.00–284.50)	0.723	3.45 (2.40–4.74)	0.544
High TG	218.00 (178.00–293.00)		3.45 (2.34–4.60)	
Normal HDL	*223.00 (186.00–293.75)*	*0.059*	3.43 (2.38–4.53)	0.254
Low HDL	*213.00 (183.00–278.00)*		3.46 (2.39–4.92)	
Normal LDL	220.00 (184.00–280.00)	0.319	3.42 (2.26–4.60)	*0.014*
High LDL	218.50 (188.25–304.50)		3.68 (2.75–4.97)	
Normal VLDL	221.00 (186.00–284.00)	0.790	3.45 (2.40–4.71)	0.645
High VLDL	219.00 (178.75–293.00)		3.45 (2.35–4.62)	

*P*-value at ≤0.05 level are italicized

Correlation analysis, both spearman as well as in partial correlation analysis after adjustment with gender, revealed significant positive correlation between serum vitamin B-12 and HDL (Additional file 3). On the other hand, folate was found to be positively correlated with TC and LDL (p ≤ 0.05) (Additional file 3).

**Fig. 2:**
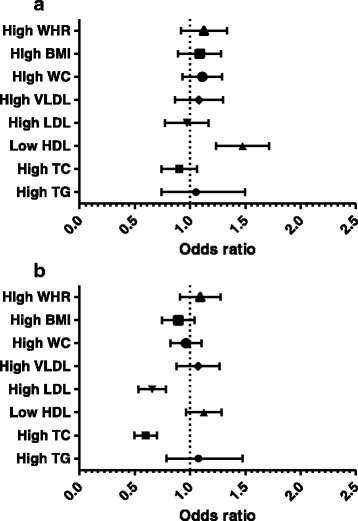
**a** Multivariate logistic regression analysis of vitamin B-12 with lipids & obesity indices. The main confounder between normal and low vitamin B-12 individuals was found to be gender, among all the general characteristics (age, gender, smoking, education, physical activity and occupation). **b** Multivariate logistic regression analysis of folate with lipids and obesity indices. The main confounder between normal and low folate individuals was found to be gender, among all the general characteristics (age, gender, smoking, education, physical activity and occupation)

Multivariate logistic regression analysis after adjustment with confounder (gender) revealed more than 1-fold increased risk of vitamin B-12 deficiency on individuals with low HDL (Fig. 2a). Except HDL, no persistent associations were found between vitamin B-12 and other lipid and also with obesity indices in all the three statistical analysis performed. Low folate was found to be significantly reducing the risk of high TC (OR-0.579, 95%CI-(0.417–0.804), *p*-value- 0.001) and high LDL (OR-0.653, 95%CI-(0.453–0.941), p-value- 0.022) in the multivariate logistic regression analysis (Fig. 2b).

## Discussion

Generally, the relationship between homocysteine and lipids (TC, TG, LDL, VLDL) were either reported positive [12, 19, 20] or were not associated [14, 21–26] as per the available literature from case-control and population based study. But only few case-control studies showed a trend of negative correlation between the variables [27]. In the present study also, a noteworthy observation was that except HDL, all the lipid indices showed a trend for negative correlation with hcy after controlling for confounders, though not significant. This was against the normal phenomenon. In order to validate our results, association of hcy with anthropometric variables of obesity (BMI, WHR, WC) were evaluated. The results were not found to be different to that of lipids. Similar to the present results, homocysteine was not associated with WC, BMI, WHR in other studies also [14, 22, 26]. The possible explanation for the inconsistency in hcy-lipid relationship with respect to biological mechanism could be that with such a high prevalence of hyper-homocysteinemia found in the studied population, it may be possible that the PEMT pathway has been disturbed and not be able to produce 30% of PC. However, the population consumes adequate food sources of choline in the form of milk & milk products, potatoes, wheat which might have helped in proper functioning of CDP-Choline or kennedy pathway producing 70% of PC and herewith preventing fat accumulation among the studied population in spite of high homocysteine.

Majorly, the positive associations of hcy-lipids, found in the literature had case-control or clinical or experimental designs which were entirely different with respect to population based study designs. In studies based on animal models, either they were knocked out for various genes or were diet-induced. On the other hand, population studies are performed on healthy individuals residing in complete natural environment, with no fortified diet or supplementation, where cultural practices such as preparation of food, amount of food intake, lifestyle and social factors are kept in observation. It may be because of this, that inconsistency in the hcy-lipid relationship is seen. Further, different population groups have different characteristics, such as ethnicity, gene pool, dietary practices and cultural & lifestyle habits, which may show variation in hcy-lipid hypothesis from population to population. Godsland IF and co-authors have mentioned about the inconsistent results of hcy-lipids relationship, and also stated that where such associations were found, they were generally found to be weak [14].

As no direct association between hcy and lipids was found, an attempt was made to evaluate if low folate and vitamin B-12 were responsible for raising homocysteine levels in this population, as these are the essential micro-nutrients of one carbon metabolic pathway.

In this population, vitamin B-12 deficiency was the main contributing factor for hyper homocysteinemia and showed stronger association than folate, as also evident in other population studies [10, 18]. Possibly, dietary deficiency of vitamin B-12 in this population might have reduced the activity of the enzyme methionine synthase (MS) which was required for the conversion of homocysteine to methionine. Accumulation of homocysteine levels is a result of low dietary intake of vitamin b-12 which gradually altered the enzymatic activity of MS.

Due to adequate intake of folate in the form of green leafy vegetables, the association of folate with HHcy was found to be weak in the study. On the other hand, the reason for high prevalence of vitamin B-12 deficiency (57.6%) in the population was their inadequate dietary intake, due to the practice of vegetarianism. This is the common reason for vit B-12 deficiency in majority of the Indian populations [10]. Besides food sources of vitamin B-12, drinking water in the form of demineralised water have less content of vitamin B-12 due to removal of cobalt and those microorganisms responsible for endogenous production of Vitamin B12 directly or indirectly [28]. The studied population mostly used either RO water or boring water for drinking. Vitamin B12 deficiency was associated with use of RO processed drinking water [28]. Other than inadequate dietary intake and usage of RO water; lack of awareness, gender difference are the other factors due to which the population could not prevent vitamin B-12 deficiency.

A direct role of dyslipidemia with vitamin B-12 and folate deficiency was also evaluated in the present study, as these micro-nutrients were found to be regulating lipid levels independently [12, 29, 30]. The results revealed an independent association of low HDL (dyslipidemia) with low vitamin B-12 (nutritional determinant of Hcy), making dietary vit B-12 level low in double dose, which was posing a high risk for hyper-homocysteinemia (cardio-vascular adversity). Further, the connection of HDL with Vit B-12 was an indication of severe under-nutrition leading to disturbance in both the metabolic pathways (homocysteine and lipid pathway) of the population. The possible mechanism for the association between vitamin B-12 deficiency and adverse lipid parameters is different. It can be said that due to vitamin B-12 deficiency in this population, the metabolic reaction involving the conversion of methylmalonyl CoA (MM-CoA) to succinyl-CoA might have been blocked. This resulted in accumulation of MM-CoA which further inhibited the function of fatty acid oxidation enzyme, carnitine palmitoyl transferase (CPT1), thus causing lipogenesis [29]. Researchers have discussed that the complex biological mechanism linking vitamin B-12 and lipids (especially HDL) is not well elucidated. The results of the present study were concordant with an African population study which also found association of low vit B-12 with low HDL [29, 31]. This can be stated that there was no sign of lipid initiated hyper-homocysteinemia (a metabolic distress) in this population but lipid (HDL) initiated nutritional deficiency (vit B-12), leading to hyper-homocysteinemia.

The studied population consumes good nutrition in the form of folate. The results showed that folate had significantly reduced risk for high LDL and high TC as against the normal phenomenon. It indicated a protective role of folate among those with high TC/LDL. Lipids were not able to increase homocysteine levels as the high levels of TC/LDL were controlled by folate in the present study. Further, understanding the biological mechanism between folate and lipids along with role of genetics is of utmost priority.

It can be thought that the population was cardio-protective because of minimal prevalence of major cardiac disorders like CAD, T2DM found (1%) but the association of vitamin B-12 with HDL is a matter of concern. The present studied population is unsafe especially in terms of under-nutrition, i.e., vitamin B-12 deficiency which is the major culprit, disturbing two metabolic pathways by associating with HDL and causing hyper-homocysteinemia.

## Conclusion

The present study does not support the complementing effect of “hcy-lipid”. Low vitamin B-12 was the main culprit, causing metabolic disturbance in both the metabolic pathways (lipid & one carbon metabolic pathway) by interacting with HDL. The results of the present study may not be generalized to other ethnic groups. However, the extrapolation of research on animal models to humans needs to be dealt with caution as both studies are studied under different conditions. Thus in countries like India where diversity at every level and every sphere is next to Africa, such population based association studies need to be encouraged to document and collect a body of evidence based on geography, ethnicity & culture to have a meaningful population based public health strategies.

### Limitations

First, the data on food frequency questionnaire (FFQ) was not collected which could have further helped in validation of the study**.** Second, Vitamin B-6 is a major determinant of high Hcy, which was not estimated in the present study. Third, the cross-sectional design of the study is one of the limitations.

## Additional files

Additional file 1: Correlation of homocysteine with lipids, and anthropometric obesity indices. (DOCX 13 kb)

Additional file 2: **Table S1.** Distribution of general characteristics between vitamin B-12 normal & vitamin B-12 deficient groups and folate normal & folate deficient groups. (DOC 108 kb)

Additional file 3: Correlation of vitamin B-12 and folate with lipids, & obesity indices. (DOCX 14 kb)

## Abbreviations

AIIMS: All India Institute of Medical Sciences
BMI: Basic metabolic Index
CAD: Coronary Artery Disease
CVDs: Cardiovascular diseases
Hcy: homocysteine
HDL: high density lipoprotein
HHcy: Hyper-homocysteinemia
LDL: Low density lipoprotein
TC: total cholesterol
TG: triglycerides
VLDL: very low density lipoprotein
WC: Waist circumference
