# Supplementary material for: Metabolic distress in lipid & one carbon metabolic pathway through low vitamin B-12: a population based study from North India
Source: Lipids Health Dis. 2018 Apr 25;17:96. doi: 10.1186/s12944-018-0748-y (PMC5918761; doi:10.1186/s12944-018-0748-y)
Supplement: Supplementary file 1 — Correlation of homocysteine with lipids, and anthropometric obesity indices. (DOCX 13 kb) [file 12944_2018_748_MOESM1_ESM.docx]

**Additional file 1 Correlation of homocysteine with lipids, and anthropometric obesity indices.**

|  | **Spearman correlation**  **(rho), P-value** | **Partial correlation (controlled for age, gender, smoking, education)** |
| --- | --- | --- |
| Hcy vs TC | 0.004, 0.891 | -0.025, 0.429 |
| Hcy vs TG | -0.013, 0.656 | -0.058, 0.068 |
| Hcy vs HDL | 0.000, 0.998 | 0.048, 0.119 |
| Hcy vs LDL | -0.010, 0.726 | -0.040, 0.206 |
| Hcy vs VLDL | -0.012, 0.679 | -0.058, 0.065 |
| Hcy vs WC | **0.085, 0.003** | 0.028, 0.368 |
| Hcy vs BMI | -0.042, 0.148 | 0.011, 0.750 |
| Hcy vs WHR | **0.141, 0.000** | 0.034, 0.283 |
| Hcy vs FOL | **-0.126, 0.000** | **-0.137, 0.000** |
| Hcy vs VIT B-12 | **-0.171, 0.000** | **-0.165, 0.000** |

**P-value at ≤0.05 level**
