# Supplementary material for: Metabolic distress in lipid & one carbon metabolic pathway through low vitamin B-12: a population based study from North India
Source: Lipids Health Dis. 2018 Apr 25;17:96. doi: 10.1186/s12944-018-0748-y (PMC5918761; doi:10.1186/s12944-018-0748-y)
Supplement: Supplementary file 2 — Table S1. Distribution of general characteristics between vitamin B-12 normal & vitamin B-12 deficient groups and folate normal & folate deficient groups. (DOC 108 kb) [file 12944_2018_748_MOESM2_ESM.doc]

**Supplementary file**

**Table (for figure 1). Multivariate logistic regression analysis of hcy with lipids obesity indices and vitamins.**

|  | **Chi-square** | **Normal HCY**  **n (%)** | **High HCY**  **n (%)** | **Unadjusted OR (95%CI), p-value** | **Adjusted OR (95%CI), p-value**  **(age, gender, smoking, education)** |
| --- | --- | --- | --- | --- | --- |
| **Normal TG** | 0.519 | 303(79.3) | 736 (80.9) | 0.907 (0.673-1.221) | 0.824 (0.591-1.150), 0.256 |
| **High TG** |  | 79 (20.7) | 174 (19.1) |  |  |
| **Normal TC** | 0.538 | 289 (74.3) | 688 (72.7) | 1.088 (0.832-1.423) | 0.897 (0.663-1.213), 0.480 |
| **High TC** |  | 100 (25.7) | 259 (27.3) |  |  |
| **Normal HDL** | 0.107 | 201 (51.8) | 533 (56.6) | 0.823 (0.649 -1.043) | 0.927 (0.710-1.209), 0.574 |
| **Low HDL** |  | 187 (48.2) | 408 (43.4) |  |  |
| **Normal LDL** | 0.118 | 316 (82.3) | 725 (78.5) | 1.276 (0.940-1.731) | 1.169 (0.827-1.654), 0.376 |
| **High LDL** |  | 68 (17.7) | 199 (21.5) |  |  |
| **Normal VLDL** | 0.476 | 305 (79.4) | 740 (81.1) | 0.897 (0.666-1.209) | 0.817 (0.585-1.139), 0.233 |
| **High VLDL** |  | 79 (20.6) | 172 (18.9) |  |  |
| **Normal WC** | 0.757 | 204 (54.4) | 495 (53.5) | 1.039 (0.816-1.322) | 1.089 (0.829-1.430), 0.540, |
| **HIGH WC** |  | 171 (45.6) | 431 (46.5) |  |  |
| **Normal BMI** | 0.412 | 183 (59.6) | 403 (56.8) | 1.121 (0.854-1.471) | 1.159 (0.856-1.569), 0.340 |
| **HIGH BMI** |  | 124 (40.4) | 306 (43.2) |  |  |
| **Normal WHR** | 0.959 | 92 (25.0) | 230 (25.1) | 0.993 (0.751-1.313) | 1.128 (0.817-1.556), 0.465 |
| **HIGH WHR** |  | 276 (75.0) | 685 (74.9) |  |  |
| **Normal FOL** | 0.504 | 214 (66.5) | 520 (64.4) | 1.097 (0.836-1.441) | 1.054 (0.777-1.430), 0.735 |
| **Low FOL** |  | 108 (33.5) | 288 (35.6) |  |  |
| **Normal VIT B-12** | 0.000 | 151 (59.9) | 269 (46.0) | **1.756 (1.301-2.370)** | **1.813 (1.296-2.538), 0.001** |
| **Low VIT B-12** |  | 101 (40.1) | 316 (54.0) |  |  |

**P-value at ≤0.05 level**

**Table (for figure 2a). Multivariate logistic regression analysis of folate with lipids & obesity indices**

|  | **Chi-square** | **Normal Folate**  **(%)** | **Low folate**  **(%)** | **Adjusted OR (95%CI), p-value**  **(gender only )** |
| --- | --- | --- | --- | --- |
| **Normal TG** | 0.527 | 64.7 | 35.3 | 1.076 (0.785-1.475), 0.648 |
| **High TG** |  | 62.3 | 37.7 |  |
| **Normal TC** | 0.001 | 61.9 | 38.1 | **0.580 (0.430-0.784), 0.000** |
| **High TC** |  | 72.6 | 27.4 |  |
| **Normal HDL** | 0.647 | 64.6 | 35.4 | 1.102 (0.859-1.413), 0.445 |
| **Low HDL** |  | 63.2 | 36.8 |  |
| **Normal LDL** | 0.016 | 62.6 | 37.4 | **0.633 (0.453-0.887), 0.008** |
| **High LDL** |  | 71.6 | 28.4 |  |
| **Normal VLDL** | 0.683 | 64.4 | 35.6 | 1.038 (0.756-1.426), 0.816 |
| **High VLDL** |  | 62.9 | 37.1 |  |
| **Normal WC** | 0.432 | 63.4 | 36.6 | 0.943 (0.733-1.213), 0.648 |
| **HIGH WC** |  | 65.7 | 34.3 |  |
| **Normal BMI** | 0.296 | 63.4 | 36.6 | 0.870 (0.653-1.159), 0.342 |
| **HIGH BMI** |  | 66.9 | 33.1 |  |
| **Normal WHR** | 0.967 | 64.1 | 35.9 | 1.062 (0.792-1.424), 0.688 |
| **HIGH WHR** |  | 64.2 | 35.8 |  |

**Table (for figure 2b). Multivariate logistic regression analysis of vitamin B-12 with lipids & obesity indices**

|  | **Chi-square** | **Normal Vit B-12 (%)** | **Low Vit B-12**  **(%)** | **Adjusted OR (95%CI), p-value**  **(gender only )** |
| --- | --- | --- | --- | --- |
| **Normal TG** | 0.504 | 50.8 | 49.2 | 1.054 (0.743-1.496), 0.767 |
| **High TG** |  | 47.8 | 52.2 |  |
| **Normal TC** | 0.644 | 50.2 | 49.8 | 0.873 (0.639-1.194), 0.396 |
| **High TC** |  | 52.1 | 47.9 |  |
| **Normal HDL** | 0.054 | 53.0 | 47.0 | **1.436 (1.079-1.909), 0.013** |
| **Low HDL** |  | 46.1 | 53.9 |  |
| **Normal LDL** | 0.888 | 50.4 | 49.6 | 0.931 (0.650-1.332), 0.694 |
| **High LDL** |  | 51.0 | 49.0 |  |
| **Normal VLDL** | 0.586 | 50.8 | 49.2 | 1.037 (0.730-1.474), 0.837 |
| **High VLDL** |  | 48.4 | 51.6 |  |
| **Normal WC** | 0.941 | 50.8 | 49.2 | 1.083 (0.818-1.434), 0.579 |
| **HIGH WC** |  | 50.5 | 49.5 |  |
| **Normal BMI** | 0.950 | 50.1 | 49.9 | 1.052 (0.768-1.442), 0.752 |
| **HIGH BMI** |  | 50.4 | 49.6 |  |
| **Normal WHR** | 0.801 | 50.0 | 50.0 | 1.087 (0.785-1.506), 0.615 |
| **HIGH WHR** |  | 51.0 | 49.0 |  |

**P-value at ≤0.05 level**

**Table S1: Distribution of general characteristics between vitamin B12 normal & vitamin b12 deficient groups and folate normal & folate deficient groups.**

|  | **Normal vit B-12** | **Low vit B-12** | **Chi-Square, p-value** | **Normal Folate** | **Low folate** | **Chi-Square, p-value** |
| --- | --- | --- | --- | --- | --- | --- |
| **Age (Median, in years)** | 45.00 | 47.00 | 0.215 **(Mann whitney test)** | 46.00 | 46.00 | 0.986 **(Mann whitney test)** |
| **Smoking**  Smokers (%) | 49.4 | 47.5 | 0.588 | 52.9 | 56.1 | 0.318 |
| Non smokers (%) | 50.6 | 52.5 |  | 47.1 | 43.9 |  |
| **Gender**  Males (%) | 23.3 | 33.3 | **0.001** | 29.5 | 35.5 | **0.036** |
| Females (%) | 76.7 | 66.7 |  | 70.5 | 64.5 |  |
| **Education**  Illiterate (%) | 59.3 | 57.7 | 0.653 | 60.2 | 54.9 | 0.104 |
| Literate (%) | 40.7 | 42.3 |  | 39.8 | 45.1 |  |
| **Physical activity**  Sedentary (%) | 16.5 | 13.5 | 0.290 | 15.3 | 15.7 | 0.872 |
| Active (%) | 83.5 | 86.5 |  | 84.7 | 84.3 |  |
| **Occupation**  Government employees + Business persons + others (%) | 9.7 | 12.9 | 0.154 | 11.3 | 14.1 | 0.179 |
| Agricultural worker + Housewives (%) | 90.3 | 87.1 |  | 88.7 | 85.9 |  |

**P-value at ≤0.05 level**
