# Supplementary material for: Metabolic distress in lipid & one carbon metabolic pathway through low vitamin B-12: a population based study from North India
Source: Lipids Health Dis. 2018 Apr 25;17:96. doi: 10.1186/s12944-018-0748-y (PMC5918761; doi:10.1186/s12944-018-0748-y)
Supplement: Supplementary file 3 — Correlation of vitamin B-12 and folate with lipids, & obesity indices. (DOCX 14 kb) [file 12944_2018_748_MOESM3_ESM.docx]

**Additional file 3 Correlation of vit B-12 and folate with lipids, & obesity indices.**

|  | **Vitamin B-12**  **Spearman correlation (rho), P-value** | **Vitamin B-12 partial**  **correlation (r), P-value**  **(adjusted for only gender )** | **Folate Spearman correlation (rho), P-value** | **Folate partial correlation (r), P-value (adjusted for only gender)** |
| --- | --- | --- | --- | --- |
| **TC** | **0.059, 0.085** | **0.078, 0.024** | **0.107, 0.000** | **0.104, 0.000** |
| **TG** | -0.047, 0.185 | -0.005, 0.879 | **-0.074, 0.015** | **-0.060, 0.050** |
| **HDL** | **0.112, 0.001** | **0.074, 0.034** | -0.011, 0.704 | -0.032, 0.284 |
| **LDL** | 0.041, 0.235 | 0.047, 0.179 | **0.134, 0.000** | **0.130, 0.000** |
| **VLDL** | -0.043, 0.215 | -0.007, 0.852 | **-0.065, 0.031** | -0.052, 0.086 |
| **WC** | 0.005, 0.891 | 0.039, 0.271 | -0.038, 0.206 | -0.036, 0.235 |
| **BMI** | -0.003, 0.925 | 0.012, 0.730 | 0.019, 0.530 | -0.007, 0.822 |
| **WHR** | -0.055, 0.116 | 0.006, 0.872 | 0.003, 0.923 | 0.012, 0.701 |

**P-value at ≤0.05 level**
